# Gait in Children with Attention-Deficit Hyperactivity Disorder in a Dual-Task Paradigm

**DOI:** 10.3389/fpsyg.2017.00034

**Published:** 2017-01-19

**Authors:** Olivia Manicolo, Alexander Grob, Priska Hagmann-von Arx

**Affiliations:** Department of Psychology, University of BaselBasel, Switzerland

**Keywords:** ADHD, gait, dual-task, executive functions, children

## Abstract

The aim was to examine gait in school-aged children with attention-deficit hyperactivity disorder (ADHD) and typically developing controls in a dual-task paradigm. Thirty children with ADHD (without or off medication) aged 7–13 years and 28 controls walked without an additional task (single-task walking) and while performing a concurrent cognitive or motor task (dual-task walking). Gait was assessed using GAITRite recordings of spatiotemporal and variability gait parameters. Compared to single-task walking, dual-tasking significantly altered walking performance of children with and without ADHD, whereby dual-task effects on gait were not different between the two groups. For both children with ADHD and controls the motor concurrent task had a stronger effect on gait than the cognitive concurrent task. Gait in children with and without ADHD is affected in a dual-task paradigm indicating that walking requires executive functions. Future investigations of children's dual-task walking should account for the type of concurrent tasks.

## Introduction

Attention-deficit hyperactivity disorder (ADHD) is one of the most frequently diagnosed disorders in school-aged children with a prevalence of 5–10% (Polanczyk and Rhode, 2007). It is characterized by three core symptoms: Impulsivity, hyperactivity, and inattention (American Psychiatric Association, 2013) and has repeatedly been linked to deficits in executive functions (e.g., Steger et al., 2001; Gillberg, 2003; Wilding, 2005; Willcutt et al., 2005). Furthermore, children diagnosed with ADHD are at higher risk for fine and gross motor impairments (Kaiser et al., 2015). Regarding gross motor skills, they are often described as clumsy, having poor motor coordination, and showing more difficulties in static and dynamic balance than typically developing children (Racine et al., 2008; Shum and Pang, 2009; Fliers et al., 2010). Additionally, children with ADHD are more likely to show poor performance in physical education classes and are more prone to injuries, such as being struck by an object (Pastor and Reuben, 2006).

However, while many studies investigated motor performance including movement skills, motor coordination, balance as well as physical fitness in children with ADHD (e.g., Harvey and Reid, 1997, 2003; Piek et al., 1999; Raberger and Wimmer, 2003; Buderath et al., 2009), far less is known regarding their walking pattern, a functionally highly relevant aspect of motor performance. Typically developing children establish a mature walking pattern at about 7 years (Adolph et al., 2003). However, there is evidence that gait variability (i.e., stride-to-stride fluctuations reflecting the automaticity and regularity of a walking pattern) continues to develop into later childhood (Hausdorff et al., 1999; Hagmann-von Arx et al., 2016). So far, three studies have investigated spatiotemporal and variability measures of gait in school-aged children with ADHD (without or off medication) using instrumented gait analysis techniques such as electronic walkway systems or electronic footswitches (Leitner et al., 2007; Papadopoulos et al., 2014; Manicolo et al., 2016). Their results consistently indicated increased gait variability for children with ADHD compared to controls while no group differences were found for spatiotemporal gait measures such as velocity (Leitner et al., 2007; Papadopoulos et al., 2014; Manicolo et al., 2016). As increased gait variability is assumed to reflect more inconsistent stepping patterns during walking (Hausdorff, 2005), those findings indicate a less regular gait pattern of school-aged children with ADHD compared to controls.

However, in everyday life children usually do things concomitantly while walking such as listening to someone talk or fastening their jacket buttons. Previous research has shown that such dual-task situations alter children's walking performance (Huang et al., 2003; Cherng et al., 2007; Schaefer et al., 2010; Boonyong et al., 2012; Hung et al., 2013; Hagmann-von Arx et al., 2015), indicating that the regulation of gait is not a fully automatic activity but rather requires cognitive processes (Woollacott and Shumway-Cook, 2002). Dividing attention between two tasks in dual-task situations is considered an executive function task (Springer et al., 2006) and places demands on the central executive (Karatekin, 2004). The central executive is part of the working memory model proposed by Baddeley and Hitch (1974) and is seen as a supervisory system that coordinates and controls cognitive processes. The working memory model further posits temporary buffers that hold either verbal or spatial information and postulates rehearsal mechanisms that maintain this information in mind. The central executive coordinates the activities of the verbal and spatial buffer systems and controls attention (Baddeley and Hitch, 1974). Thus, a measure of the central executive is the ability to do two things at the same time as required in dual-task paradigms (Baddeley et al., 1997; Karatekin, 2004). The working memory in turn is proposed to form one of the core components of executive functions, which refer to a family of top-down mental processes necessary for adaptive planning of behaviors (Anderson, 2002; Diamond, 2013). Studies including clinical adult samples characterized by impaired executive functions (e.g., Alzheimer's or Parkinson's disease) reported poorer gait performance in the clinical samples compared to healthy controls in dual-task conditions (Sheridan et al., 2003; Yogev et al., 2005). Further, healthy older adults show lower performance in executive functions compared to young adults (Glisky, 2007) what in turn may negatively affect their task performance in dual-task conditions where attention needs to be divided between concurrent tasks (Beurskens and Bock, 2012; Tsang, 2013). Therefore, it is assumed that impaired executive functions contribute to stronger effects of dual-tasking on gait by limiting the ability to devote the appropriate amount of attention toward walking when simultaneously performing another task (Hausdorff, 2005).

Although ADHD has repeatedly been linked to deficits in executive functions (e.g., Steger et al., 2001; Gillberg, 2003; Wilding, 2005; Willcutt et al., 2005) as for example in the central executive component of the working memory (e.g., Karatekin, 2004), little is known regarding the role that those limitations may play in dual-task gait in children with ADHD. To our knowledge, so far one study has investigated walking patterns of school-aged children with ADHD in a dual-task paradigm. Leitner et al. (2007) measured gait in a dual-task condition where children were instructed to walk and simultaneously listen to a text on tape and count how many times a keyword appeared. Within-group comparisons showed that children with ADHD as well as controls walked with reduced velocity and a tendency toward increased stride time in the dual-task condition compared to normal walking, implying that in both groups gait requires executive functions. Furthermore, between-group comparisons showed that in the dual-task condition both groups walked with similar gait variability, velocity, and stride time. Therefore, the effect of dual-tasking on gait was comparable between children with and without ADHD, although it may have been assumed that children with ADHD would show lower gait performance (i.e., lower gait velocity and higher gait variability) compared to controls when their impaired executive functions are additionally taxed by a concurrent task (Leitner et al., 2007). However, the sample studied by Leitner et al. (2007) was rather small, limiting the power of these analyses.

Investigations of dual-task effects on gait among typically developing children showed that gait alterations are apparent for both motor and cognitive concurrent tasks and that effects on walking may differ between the two types of concurrent tasks. For example, Cherng et al. (2007) investigated school-aged children while they walked and concurrently performed an easy or a difficult motor (carrying a tray with or without marbles on it) or cognitive (repeating a series of digits forward or backward) task. Compared to single-task walking, both cognitive concurrent tasks as well as the difficult motor concurrent task caused significant gait alterations. Additionally, results showed that the difficult motor concurrent task led to greater gait alterations (i.e., greater decrease in stride length and greater increase in double limb support) compared to the cognitive concurrent tasks. In a similar vein, a recent study conducted by Hagmann-von Arx et al. (2016) including school-aged children confirmed these findings by showing that gait performance was stronger affected in a motor dual-task condition in which children were asked to fasten and unfasten a shirt button than in a cognitive dual-task condition in which children were asked to listen to and memorize digits while walking.

These findings may be interpreted from the perspective of the multiple-resource model of attention (Wickens, 1991) assuming that two tasks will interfere with each other if they share the same pool of resources. Hence, a cognitive concurrent task might not cause the same amount of gait alterations as a motor concurrent task that shares resources with walking (Yogev-Seligmann et al., 2008). To our knowledge, this assumption has so far not been investigated in children with ADHD since Leitner et al. (2007) included a cognitive concurrent task while walking (i.e., listen to a text and count a key word) but no motor concurrent task.

The main goal of this study was to investigate gait in children with and without ADHD at school-age when children have reached an adult-like gait pattern (Adolph et al., 2003) but variability gait parameters are still developing (Hausdorff et al., 1999; Hagmann-von Arx et al., 2016) in a dual-task paradigm including both a concurrent cognitive and motor task condition. First, we expected that a concurrent cognitive and motor task would negatively affect gait performance of children with and without ADHD as there is evidence that gait requires executive functions (e.g., Woollacott and Shumway-Cook, 2002). Second, we expected to find more strongly compromised dual-task gait performance in children with ADHD compared to children without ADHD due to their impaired executive functions (e.g., Willcutt et al., 2005; Beurskens and Bock, 2012; Tsang, 2013). Finally, we expected to find a stronger dual-task effect on gait for the motor concurrent task compared to the cognitive concurrent task in children with and without ADHD drawing on the assumption that tasks sharing the same pool of processing resources interfere with each other more strongly (Yogev-Seligmann et al., 2008).

## Materials and methods

### Participants

The sample included in this study comprised 30 children with ADHD (25 boys and 5 girls, *M*_*age*_ = 10.9 years, age range: 7–13 years, without or off medication) and 28 typically developing controls (25 boys and 3 girls, *M*_*age*_ = 10.8 years, age range: 7–13 years) and has been described in a previous study in detail (Manicolo et al., 2016). Children with ADHD were diagnosed according to the DSM-IV or ICD-10 by privately practicing pediatricians and pediatricians at the University Children's Hospital Basel. The diagnosis was confirmed by parental ratings using the Conners' Parent Rating Scale (Conners, 2001). Twenty-one children with ADHD were on stimulant medication (e.g., Methylphenidate, Ritalin®, Novartis Pharmaceuticals Corporation) and discontinued medication at least 24 h before testing following recommendations by Thompson (2007). Children without ADHD were recruited from local schools and screened for ADHD symptoms using the Conners' Parent Rating Scale (Conners, 2001). No control child showed symptoms of ADHD. Children with and without ADHD did not differ in demographics (i.e., age, sex, height, weight, leg length; Table 1). None of the 58 included children were at risk for developmental coordination disorder (DCD) (i.e., >16th percentile in the German version of the Movement Assessment Battery for Children 2nd edition) (Petermann, 2008) or intellectual impairment (i.e., IQ > 70 assessed by the German version of the Wechsler Intelligence Scale for Children 4th edition) (Petermann and Petermann, 2011). The Ethics Committee of Basel approved the study protocol. Parents gave written informed consent for the children to participate and assent was obtained from the child.

**Table 1:** **Demographic characteristics of children with attention-deficit hyperactivity disorder (ADHD) and controls**.

**Characteristic**	**ADHD (***n*** = 30)**	**Controls (***n*** = 28)**	***p***
Age (years)	10.9 (1.4)	10.8 (1.4)	0.847
Sex (girls:boys)	5:25	3:25	0.512
Height (cm)	142.7 (29.7)	140.3 (29.3)	0.761
Weight (kg)	40.4 (16.8)	36.8 (12.0)	0.361
Leg length (cm)^a^	75.4 (16.6)	74.7 (16.9)	0.862

*Data are mean (SD) or number; p-values are given for independent t-test, or χ^2^ test*.

a *Leg length was measured with footwear from greater trochanter to the floor, bisecting the lateral malleolus*.

The previous study (Manicolo et al., 2016) showed, that regarding single-task walking there were no group differences in velocity (*p* = 0.769, *d* = 0.08) and stride length variability (*p* = 0.280, *d* = 0.44), whereas children with ADHD walked with higher stride time variability than children without ADHD (*p* = 0.012, *d* = 0.50). In the present study we additionally included the gait parameters stride length, step length, stride time, as well as stride velocity variability, and step length variability (see Table 2). While the groups did not differ in stride length (*p* = 0.571, *d* = 0.15), step length (*p* = 0.529, *d* = 0.21), and stride time (*p* = 0.291, *d* = 0.23), children with ADHD showed significantly higher stride velocity variability (*p* = 0.012, *d* = 0.68) and step length variability (*p* = 0.043, *d* = 0.55) than controls in single-task walking.

**Table 2:** **Means (and standard deviations) of gait parameters for children with attention-deficit hyperactivity disorder (ADHD) and controls in single- and dual-task conditions**.

**Gait parameters**	**ADHD (***n*** = 30)**	**Controls (***n*** = 28)**
**VELOCITY (cm/s)**		
Single task	127.75 (21.76)	131.51 (15.64)
Dual task: Digits	101.62 (19.51)	103.74 (15.28)
Dual task: Button	82.57 (20.24)	88.46 (16.74)
**STRIDE LENGTH (cm)**		
Single task	130.30 (16.03)	132.47 (12.61)
Dual task: Digits	113.65 (15.14)	114.18 (11.31)
Dual task: Button	99.73 (17.30)	103.69 (15.40)
**STEP LENGTH (cm)**		
Single task	64.88 (8.24)	66.41 (6.03)
Dual task: Digits	56.42 (7.80)	57.09 (5.66)
Dual task: Button	49.00 (10.00)	51.78 (7.80)
**STRIDE TIME (s)**		
Single task	1.04 (0.10)	1.01 (0.08)
Dual task: Digits	1.13 (0.13)	1.11 (0.10)
Dual task: Button	1.25 (0.20)	1.19 (0.10)
**STRIDE VELOCITY VARIABILITY (%)**		
Single task	3.05 (1.43)	2.25 (0.83)
Dual task: Digits	3.69 (1.26)	3.64 (1.99)
Dual task: Button	5.89 (3.98)	5.21 (2.84)
**STRIDE LENGTH VARIABILITY (%)**		
Single task	2.28 (1.13)	1.88 (0.61)
Dual task: Digits	2.52 (0.97)	2.61 (1.50)
Dual task: Button	4.72 (4.00)	4.48 (3.95)
**STEP LENGTH VARIABILITY (%)**		
Single task	2.89 (0.94)	2.42 (0.76)
Dual task: Digits	3.68 (1.60)	3.52 (1.62)
Dual task: Button	7.70 (11.35)	5.76 (4.92)
**STRIDE TIME VARIABILITY (%)**		
Single-task	2.45 (1.50)	1.67 (0.55)
Dual task: Digits	2.69 (0.92)	2.48 (1.58)
Dual task: Button	4.54 (2.78)	4.02 (2.55)

*Digits, listening to and memorizing digits; Button, unfastening and fastening a button*.

### Equipment and measures

All gait analyses were performed according to the European guidelines for spatial-temporal gait analysis (Kressig et al., 2006). Gait was measured using the GAITRite electronic walkway system (GAITRite Platinum; CIR Systems, USA), a 701-cm-long walkway with 23,040 integrated pressure sensors. The validity and reliability of gait assessment using GAITRite for children is well-established (Thorpe et al., 2005). A 1.25-m non-recordable zone was added on each end of the walkway to minimize the effects of acceleration and deceleration. Hence, children walked ~10 m per walk comprising on average 8 steps. After each walk, data were analyzed using GAITRite software. The following gait parameters were evaluated: Velocity (obtained by dividing the distance traveled by ambulation time expressed in centimeters per second), stride length (the distance between the heel points of two consecutive footfalls of the same foot), step length (the distance from the heel center of the current footprint to the heel center of the previous footprint on the opposite foot), stride time (the time elapsed between the first contact of two consecutive footfalls of the same foot expressed in seconds), and gait variability, assessed as stride-to-stride variability in stride velocity, stride length and stride time, using the percentage coefficient of variation (standard deviation/mean × 100).

### Design and procedure

The concurrent tasks were administered prior to gait assessment for 10 s while children were standing (single-task condition). The concurrent tasks were selected according to previous dual-task related research and included a cognitive and a motor concurrent task. The cognitive task comprised listening to and memorizing digits (digits) (Lindenberger et al., 2000; Leitner et al., 2007), where children heard a list comprising five digits presented in a randomized order from a computer over loudspeakers that were installed at the front left and front right corner of the laboratory. Afterward, the children were asked to recall the digits and performance on this task was measured as the number of correctly recalled digits with a maximum score of five. The motor task comprised continuously unfastening and fastening a shirt button at stomach height (button) (Ebersbach et al., 1995; Yang et al., 2007) and performance was measured as the number of times the button could be unfastened and fastened.

Before the commencement of the gait measurement children were given one demonstration trial and a practice trial. Then, children performed four trials of walking at their regular pace without any additional task (single-task walking). Afterward, children walked at their regular pace while completing the concurrent cognitive (digits) or motor (button) task (dual-task conditions) with two walks in each condition. Gait parameters were averaged over the trials for further data analysis. In the dual-task conditions participants were not instructed to prioritize either one of the two tasks.

### Statistical analysis

Effects of dual-task conditions on gait were examined using repeated-measures MANOVAs with group as a between subject factor (ADHD vs. control) and walking condition as a within-subject factor (single-task walking, dual-task walking digits, dual-task walking button) for each gait parameter. Additionally, MANOVAs were performed to assess group differences in concurrent task performance during single-task condition (i.e., when children were standing) and during dual-task conditions.

Significant effects were followed up with Bonferroni corrected *post-hoc* pairwise comparisons. If an extreme value (defined as a score exceeding 3 *SD*s from the group mean) occurred in the gait parameters, scores were truncated to ± 3 *SD*. The criterion level for statistical significance was set at *p* < 0.05 and the *F* statistic, *p* value (two-tailed), and effect sizes (η^2^) are reported.

## Results

For spatiotemporal and variability measures of gait, means and standard deviations in single- and dual-task conditions are shown in Table 2.

Statistical results from the repeated-measures MANOVAs for each gait parameter are presented in Table 3. For all gait parameters, a significant within-subject effect of walking condition emerged [Wilks' multivariate test, *F*_(2, 52)_ = 17.420–278.25, *p* < 0.001, η^2^ = 0.273–0.877]. Pairwise comparisons revealed higher velocity, higher stride length, higher step length, and lower stride time in single-task walking compared to both dual-task walking conditions (*p* < 0.001), and in the dual-task walking condition digits compared to button (*p* < 0.001). For all variability measures, pairwise comparisons revealed lower gait variability in single-task walking compared to both dual-task walking conditions (*p* < 0.05), and in the dual-task condition digits compared to button (*p* < 0.05). There was no between-subject effect of group nor a significant Walking condition × Group interaction.

**Table 3:** **Statistical results from the repeated-measures MANOVAs comparing the single- to the dual-task conditions for each gait parameter**.

**Gait parameter**	**Walking condition**			**Group**^a^			**Walking condition** × **Group**		
	***F***	***p***	**η^2^**	***F***	***p***	**η^2^**	***F***	***p***	**η^2^**
Velocity	278.253	<0.001	0.840	0.876	0.354	0.016	0.265	0.768	0.005
Stride length	260.721	<0.001	0.831	0.622	0.434	0.012	0.319	0.728	0.006
Step length	211.771	<0.001	0.794	0.781	0.381	0.014	1.011	0.367	0.018
Stride time	88.62	<0.001	0.626	1.141	0.290	0.290	1.205	0.304	0.022
Stride velocity variability	28.065	<0.001	0.346	1.57	0.216	0.029	0.517	0.598	0.010
Stride length variability	19.898	<0.001	0.273	0.243	0.624	0.005	0.211	0.810	0.004
Step length variability	9.728	<0.001	0.150	1.064	0.307	0.019	0.482	0.619	0.009
Stride time variability	33.615	<0.001	0.388	2.364	0.130	0.043	0.634	0.532	0.012

a *Children with attention-deficit hyperactivity disorder vs. controls*.

For cognitive and motor concurrent task performance during single-task condition (i.e., when children were standing), the MANOVA revealed no significant group differences [Wilks' multivariate test, *F*_(2, 53)_ = 1.442, *p* = 0.246, η^2^ = 0.052] such that both groups showed similar performance in the number of correctly recalled digits (ADHD: 3.8 ± 0.8; controls: 4.2 ± 0.7) and in the number of times the button could be fastened and unfastened (ADHD: 5.1 ± 1.2; controls: 5.3 ± 1.5). However, for dual-task conditions, the MANOVA revealed a significant group difference [Wilks' multivariate test, *F*_(2, 52)_ = 3.590, *p* = 0.035, η^2^ = 0.121], with pairwise comparisons showing that controls recalled significantly more digits (4.6 ± 0.9) than children with ADHD (3.9 ± 1.2) (*p* = 0.015) whereas the two groups did not differ in the number of times the button could be unfastened and fastened while walking (ADHD: 5.3 ± 1.6; controls: 6.0 ± 1.6).

## Discussion

This study investigated gait characteristics of school-aged children with ADHD (without or off medication). Previous research has shown that school-aged children with ADHD walk with higher gait variability compared to controls (Leitner et al., 2007; Papadopoulos et al., 2014; Manicolo et al., 2016), indicating a less regular walking pattern in children with ADHD compared to typically developing children. Our study extends this research and investigated gait in school-aged children with and without ADHD while walking and concurrently performing a cognitive and a motor concurrent task.

Our results showed that dual-task effects on gait are apparent for a cognitive and a motor concurrent task: When listening to and memorizing digits and when unfastening and fastening a button while walking both children with ADHD and typically developing controls showed a decrease in velocity, step length, and stride length whereas stride time and all measures of gait variability increased compared to single-task walking. For children with ADHD our study is therefore the first to show that not only a concurrent cognitive task (Leitner et al., 2007) but also a concurrent motor task affects gait performance. Further, our results are in line with previous research showing that in typically developing children, concurrent cognitive (Huang et al., 2003; Boonyong et al., 2012) and motor tasks (Cherng et al., 2007; Hung et al., 2013) affect gait. Hence, the here reported gait alterations in dual-task conditions indicate that in children with and without ADHD gait is not a fully automatic activity but rather requires executive functions (Woollacott and Shumway-Cook, 2002).

Further, our results showed that children with ADHD and controls did not differ in any gait parameter in both dual-task conditions. This is in contrast to our hypothesis stating that children with ADHD will show more strongly compromised dual-task gait performance compared to children without ADHD. Since impaired executive functions are common for children with ADHD (e.g., Steger et al., 2001; Gillberg, 2003; Wilding, 2005; Willcutt et al., 2005) those results may to some extent contradict previous findings reporting a link between impaired executive functions and poorer gait performance in dual-task conditions among healthy older adults compared to healthy young adults (Beurskens and Bock, 2012) and among clinical adult samples compared to healthy controls (Sheridan et al., 2003; Yogev et al., 2005). However, our results are in line with Leitner et al. (2007) reporting that the effect of dual-tasking on gait was comparable between children with and without ADHD. In this regard it has to be noted that both Leitner et al. (2007) as well as our study included a large age range by investigating participants aged between 9 and 16 years (Leitner et al., 2007) and between 7 and 13 years, respectively. This is an age range in which an adult-like gait pattern has been established (e.g., Adolph et al., 2003), but gait variability parameters continue to develop (Hausdorff et al., 1999; Hagmann-von Arx et al., 2016). Thus, this age range gives insights into different aspects of gait development. However, even though typically developing school-aged children show a mature gait pattern when normal walking (i.e., walking on flat ground at self-selected pace), they may still demonstrate challenges when altering their gait to meet dual-task constraints. For example, Gill (2015) showed that 5- to 7-year old children demonstrated more difficulties when walking with time constraints (i.e., walking to an audio metronome) compared to adults and that younger children demonstrated more difficulties compared to older children. This is in line with other studies showing that in motor tasks performance variability decreases as a function of age (Deutsch and Newell, 2005; Getchell, 2006). Thus, as our study included a large age range across childhood, it might be possible that our results are to some extent related to age-dependent variability of children's ability to alter their gait to meet constraints. Further, dual-task gait is considered as a task requiring executive functions (Springer et al., 2006). Previous studies found that children with ADHD show a maturational delay in their executive functions such that group differences in executive functions between children with and without ADHD are most evident during preschool and school age and become weaker toward adolescence (Barkley, 1997; Drechsler et al., 2005; Romine and Reynolds, 2005; Jurado and Rosselli, 2007; Hampel et al., 2009). Thus, future studies might investigate more homogenous age groups and further analyze whether group differences between children with and without ADHD regarding dual-task effects on gait become evident or are more pronounced in younger children compared to older age groups.

Furthermore, although dual-task walking itself is considered a task requiring executive functions (e.g., Karatekin, 2004; Springer et al., 2006), it is important to note that in the present study the concurrent tasks themselves did not directly challenge executive functions. The cognitive task used in this study (i.e., listening to and memorizing digits) is rather similar to a forward digit span task which is commonly used to measure short term memory. An executive function task would rather be seen in, for example, a working memory task which requires holding information in mind and working with it (Diamond, 2013). Thus, future studies might consider cognitive concurrent tasks which more strongly challenge components of executive functions such as working memory in order to analyse whether concurrent tasks like backward digit span or ordering numbers in ascending order might lead to group differences between children with and without ADHD in dual-task walking.

Regarding concurrent task performance we found that children with ADHD showed similar performance as controls in recalling digits when standing but recalled significantly fewer digits correctly than controls in the dual-task walking condition. However, when using the walkway system GAITRite, the walking time for one trial is limited. Therefore, in the dual-task condition digits the maximum amount of correctly recalled digits was five digits for each trial. Control children achieved a mean of correctly recalled digits close to the maximum, what may point to ceiling effects in the concurrent task performance that in turn might have muted group differences in this task. For the task performance in fastening and unfastening a button the two groups did neither differ in the number of how many times they fastened and unfastened a button while standing nor while simultaneously walking. Hence, for children with ADHD, the dual-task condition digits may have been more challenging than the dual-task condition button and therefore resulting in degraded performance in the concurrent task digits compared to controls. However, we were not able to directly test this assumption. Therefore, further research is needed in order to investigate the comparability of the difficulty of the concurrent tasks used in dual-task paradigms.

Finally, our results showed that dual-task effects on walking differed between the two types of concurrent tasks: For children with ADHD as well as for controls the concurrent motor task in which children had to unfasten and fasten a button caused a greater decrease in velocity and stride length, and a greater increase in stride time and gait variability than the concurrent cognitive task in which children had to listen to and memorize digits. This finding indicates that a concurrent motor task may cause greater dual-task gait decrements than a cognitive concurrent task what is in line with previous research (Cherng et al., 2007; Hagmann-von Arx et al., 2016) and may be interpreted from the perspective of the multiple-resource model of attention (Wickens, 1991). The model assumes that attentional resources are divided into various pools depending, for example, on the modality of input and response. Walking requires visual input and further involves the response of moving and controlling body segments, which Cherng et al. (2007) subsumed under the term somatosensation. The motor concurrent task button requires visual input and somatosensory response whereas the concurrent task digits requires auditory input and vocal response. Following the models' assumption, the motor concurrent task button therefore interferes more strongly with walking regarding processing resources than the cognitive concurrent task digits. This may explain why the dual-task effect on gait was greater for the concurrent motor task compared to the cognitive concurrent task in our study.

Our study has strengths and limitations. We consider it a strength that gait characteristics were assessed using the GAITRite system, which has proved to be a valid method of measuring gait parameters in children and offers the possibility of reliably identifying subtle changes in gait (Thorpe et al., 2005). During gait assessment children were wearing their normal clothes and shoes and it was therefore possible to assess gait performance as it is exhibited under daily circumstances. Furthermore, all participating children were screened for DCD in order to exclude children with significant motor deficits, which could have interfered with their gait performance. However, up to 47% of children with ADHD meet diagnostic criteria for DCD (Tervo et al., 2002; Kadesjö and Gillberg, 2003; Martin et al., 2010) and our results may not be generalized to individuals with a co-morbid diagnosis. Additionally, children with ADHD were not classified according to any ADHD subtype and it therefore remains subject to future research to investigate whether gait characteristics differ according to a particular ADHD subtype. Furthermore, future research should include further types of concurrent tasks when investigating gait of children with ADHD in dual-task conditions because previous research with typically developing children showed interference effects on gait for visual and auditory concurrent tasks (Huang et al., 2003). Finally, we analyzed temporal and spatial gait parameters as the walkway system GAITRite does not allow the capture of other gait characteristics. Hence, future studies on dual-task walking might investigate further aspects of children's gait as for example kinetic gait parameters (Chester et al., 2006).

In conclusion, the results of this study indicate that school-aged children with and without ADHD have difficulty in maintaining their usual walking performance while carrying out a concurrent cognitive or motor task, indicating that walking requires executive functions. Although ADHD has repeatedly been linked to deficits in executive functions (e.g., Steger et al., 2001; Gillberg, 2003; Wilding, 2005; Willcutt et al., 2005), these deficits did not lead to a more strongly compromised gait pattern in dual-task walking in children with ADHD compared to controls. Finally, we found dual-task gait decrements to be larger when walking and concurrently performing a motor compared to a cognitive task. Therefore, our results underscore the importance of taking the type of concurrent task into account when investigating children's gait in a dual-task paradigm. Knowing the effects concurrent tasks may have on the walking performance of children may raise the awareness of how activities should be structured in order to minimize dual-task interference and therefore possibly avoiding accidental injuries. Nonetheless, it remains to be further investigated how the here reported findings for gait in children with and without ADHD in dual-task paradigms extend to other tasks of children's everyday life.

## Author contributions

OM contributed to the study design, acquisition, analysis, and interpretation of data. Drafted and revised the manuscript, gave final approval, and agree to be accountable for all aspects of the work in ensuring that questions related to the accuracy or integrity of any part of the work are appropriately investigated and resolved. AG revised the manuscript, gave final approval, and agrees to be accountable for all aspects of the work in ensuring that questions related to the accuracy or integrity of any part of the workare appropriately investigated and resolved. PH contributed to the study design and interpretation of data, revised the manuscript, gave final approval, and agrees to be accountable for all aspects of the work in ensuring that questions related to the accuracy or integrity of any part of the work are appropriately investigated and resolved.

### Conflict of interest statement

The authors declare that the research was conducted in the absence of any commercial or financial relationships that could be construed as a potential conflict of interest.
